# Flying Firemen and Underwater Croquet 

**DOI:** 10.3201/eid3101.AC3101

**Published:** 2025-01

**Authors:** Reginald Tucker, Barbara Segal, Byron Breedlove

**Affiliations:** Centers for Disease Control and Prevention, Atlanta, Georgia, USA

**Keywords:** Jean-Marc Côté, La Chasse aux Microbes, France En L’An 2000, Exposition Universelle of 1900, flying firemen and underwater croquet, technological advances, pathogens, antimicrobial resistance, about the cover, art and science

**Figure:**
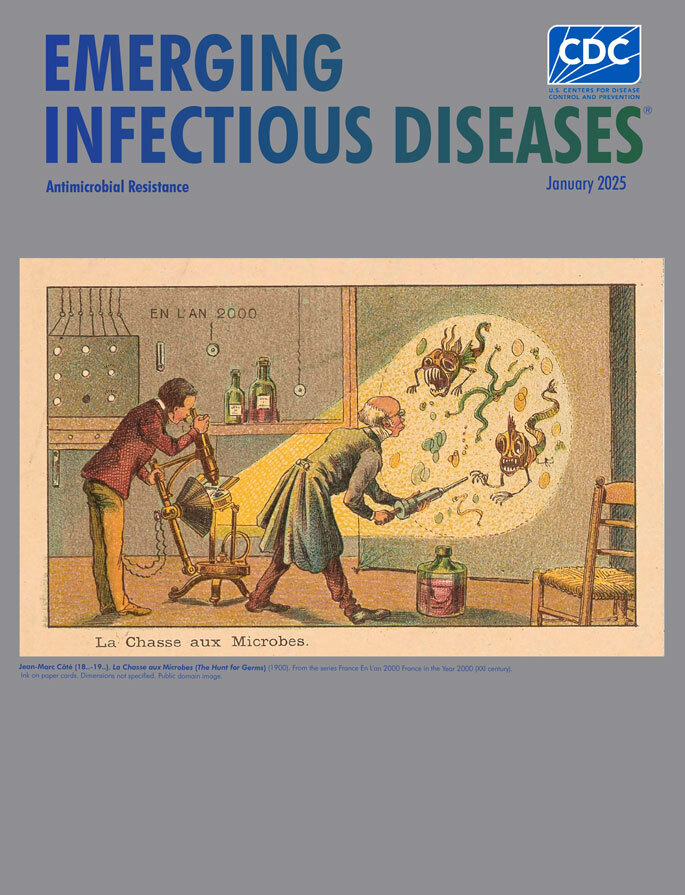
**Jean-Marc Côté (18..–19..), *La Chasse aux Microbes *(*The Hunt for Germs*) (1900)**. From the series France En L'an 2000 France in the Year 2000 (XXI century). Ink on paper cards. Dimensions not specified. Public domain image.

Predictions of future technological advances and their effects on public health have often been the subject of speculative fiction. Writers and artists alike have tried to guess what the future of disease prevention and treatment will be like and the effects that new discoveries will have on the population. Speculations often range from bleakly dystopian, in which pathogens wipe out millions of people, restructuring society; to wildly utopian, in which humans win what those artistic visionaries saw as a war against illness and disease. The promise of a utopian future was evident during the 1900 Paris Exposition.

The Exposition Universelle of 1900 was held in Paris, France, from April through November. Also known as the 1900 Paris Exposition, that world’s fair was a celebration of the artistic and technological advances of the past century and an exhibition to predict and inspire future developments. The fair showcased several technological advances, such as the Rue de l’Avenir, an electrical-powered moving sidewalk, and the Palais de l’électricité, a building decorated with electrical lightbulbs. More than 50 million people visited that exposition (the population of France at that time was around 40 million).

In 1900, Armand Gervais et Cie, a toy company that manufactured novelties, commissioned the illustrator Jean-Marc Côté to create a series of postcards celebrating the exposition. The cards were printed but never sold during the fair because of the death of Armand Gervais and the folding of his company. Seemingly lost forever, the cards were eventually acquired in the 1920s by a Parisian antique dealer. Over subsequent decades, the cards made their way to the shop Editions Renaud on the Left Bank and were sold to novelist Christopher Hyde. In 1978, Hyde shared the postcards with popular science fiction novelist Isaac Asimov. 

Côté’s postcards, *France En L’An 2000 *(*France in the Year 2000*), presented somewhat whimsical predictions of scientific advances by the year 2000. Those illustrations were an inspiration for Asimov’s 1986 book *Futuredays: A Nineteenth Century Vision of the Year 2000*, in which the postcards were first published. Asimov discussed the fantastic nature of the illustrations while contrasting the farfetched predictions, such as the card, *Ariel Firemen*, on which winged fire fighters extinguish a building fire, with the surprisingly accurate ones like *A Torpedo Plane*, on which pilots fire missiles from an airplane. 

This month’s cover highlights Côté’s postcard* Chasse aux Microbes* (*The Hunt for Germs*). It depicts 2 scientists examining monstrous-looking microbes. One scientist studies the creatures under a microscope, which is rigged to project an enlarged image of what he sees on a screen. The second scientist, who has used a large syringe to extract liquid from a jug and place a sample on the slide, leans toward the image, perhaps unsure whether to recoil or keep staring. It is also plausible that some might see the image differently and imagine that the scientist with the syringe is poised to inject an antimicrobial agent onto the projected image.

Although Côté may have missed the mark when predicting just how far instrumentation would eventually advance, he was correct when predicting that we would be able to project micrographic images in such a fashion that we could study them in detail. Asimov noted that Côté’s postcards were partially inspired by the illustrated works of the science fiction writer Jules Verne (1828–1905). In 1879, Verne published a science fiction novel, *The 500 Million of the Begums* (or alternatively *The Begum’s Millions*), which speculated about advances for preventing antimicrobial resistance through rigorous hygiene practices.

*The Begum’s Millions* tells the story of a French doctor and a German chemist, heirs of a fortune, who used their newfound wealth to build model cities in America. The chemist created a city of industry called Steel City, while the doctor established France-Ville, a city built on the principles of hygiene and the pursuit of a healthy lifestyle. Verne described the denizens of France-ville as having an imperative: “To clean, clean ceaselessly, to destroy as soon as they are formed those miasmas which constantly emanate from a human collective, such is the primary job of the central government.” That dedication to hygiene granted the residents of France-Ville a utopian level of health, while Steel City inevitably destroyed itself.

The theme of human relationships with microbial pathogens would be repeated through science fiction forever after. Inspired by Verne’s writings, Côté’s *France En L’An 2000* predicted a year 2000 full of wonder and hope, in which deep sea divers play croquet at the bottom of the sea and scientists battle microbes revealed on a projector screen. The reality is more stark. According to the Centers for Disease Control and Prevention, in the United States, more than 2.8 million antimicrobial-resistant infections occur annually, resulting in more than 35,000 deaths. Antimicrobial resistance is a high priority global health threat, associated with nearly 5 million deaths worldwide in 2019, according to a 2022 article published in *The Lancet*. Historic and contemporary artistic and literary works of speculative fiction may inspire new public health strategies for containing and responding to public health threats, including the global problem of antimicrobial resistance.
